# Exploring the relationship between proactive e-alcohol therapy and symptoms of anxiety or/and depression: Post-hoc analyses from a randomized controlled trial

**DOI:** 10.1016/j.abrep.2024.100576

**Published:** 2024-12-05

**Authors:** Kia Kejlskov Egan, Veronica Pisinger, Ulrik Becker, Janne Schurmann Tolstrup

**Affiliations:** a National Institute of Public Health, University of Southern Denmark, DK-1455 Copenhagen, Denmark

**Keywords:** Alcohol, Alcohol treatment, Anxiety and depressive symptoms, Comorbidity, E-health, Online

## Abstract

**Background:**

Individuals with problematic alcohol use often face anxiety and depressive symptoms, which can hinder treatment engagement, compliance, and effectiveness. Psychosocial therapy through video conference (e-alcohol therapy) may reduce these barriers. We explored whether 1) anxiety or/and depressive symptoms modify the effect of proactive e-alcohol therapy on treatment initiation, compliance, and alcohol intake, 2) proactive e-alcohol therapy impacts anxiety or/and depressive symptoms compared to standard care.

**Methods:**

Participants with problematic alcohol use were recruited online and randomly assigned to proactive e-alcohol therapy or standard care. Problematic alcohol use was defined by an 8+ score on the Alcohol Use Disorders Identification Test. Anxiety and depressive symptoms were measured using the Patient Health Questionnaire-4.

**Results:**

356 individuals participated; 133 showed moderate-severe symptoms of anxiety and depression at baseline. There were no significant differences between individuals with and without moderate-severe anxiety or/and depressive symptoms in the effect of proactive e-alcohol therapy versus standard care on treatment initiation (3 mo: p = 0.64; 12 mo: p = 0.97), compliance (3 mo: p = 0.40; 12 mo: p = 0.58), or alcohol intake (3 mo: p = 0.86; 12 mo: p = 0.90). No significant differences were found in the proportion of participants with moderate-severe anxiety and depressive symptoms between the two intervention groups after 3 months (OR 0.6; 95 % CI 0.3 to 1.4; p = 0.27).

**Conclusions:**

We found no evidence that anxiety or/and depressive symptoms modify the effect of proactive e-alcohol therapy on treatment initiation, compliance, or alcohol intake. Proactive e-alcohol therapy matched standard care in reducing anxiety or/and depressive symptoms over a 3-month follow-up.

## Highlights

• Affective symptoms did not modify the effect of proactive e-alcohol therapy.
• Proactive e-alcohol therapy and standard care similarly reduced affective symptoms.
• Proactive e-alcohol therapy can reduce problematic alcohol use *and* affective symptoms.

## 1 Introduction

Problematic alcohol use is one of the leading risk factors for population health worldwide (WHO, 2018) and it often co-occurs with anxiety and depression (Glantz et al., 2020). The lifetime prevalence of Alcohol Use Disorder (AUD) is 8.6 %, with 27.6 % of individuals with AUD also experiencing an anxiety disorder and 19.5 % a depressive disorder. Conversely, around one-third of individuals experiencing either anxiety or depression have struggled with AUD (Glantz et al., 2020). Co-occurring AUD and mental illness are coupled with increased symptom severity, somatic complications, and impaired social functioning (Burns et al., 2005, Lai et al., 2015). Evidence suggests a bidirectional linkage between problematic alcohol use and anxiety/depression (Boden and Fergusson, 2011, Kushner et al., 2000) as alcohol use increases the risk of depression and anxiety through neurophysiological and metabolic changes (Boden & Fergusson, 2011) and individuals with existing anxiety or depression may use alcohol as self-medication (Kushner et al., 2000).

Few individuals with problematic alcohol use engage in alcohol treatment (Degenhardt et al., 2017). In Denmark, treatment is mainly organized in outpatient clinics, posing several barriers: Geographical distance to a clinic, clinic opening hours, and attendance for face-to-face therapy, all of which are further challenged by the massive prejudice and stigmatization surrounding alcohol problems and treatment (Hammarlund et al., 2018, Schuler et al., 2015). If people also struggle with anxiety/depression, these barriers may be further complicated and relevant. Also, co-occurring anxiety and depression have been associated with poor treatment outcomes (Baker et al., 2012; Burns et al., 2005).

The core component in evidence-based alcohol treatment is psychosocial therapy founded on a combination of motivational interviewing and cognitive behavioural therapy (face-to-face alcohol therapy) (NICE, 2011, Tan et al., 2023). E-alcohol therapy is psychosocial therapy delivered via video conference. It mirrors face-to-face alcohol therapy by allowing client and therapist to see and hear each other in real-time over the internet. E-alcohol therapy has the potential to overcome geographical and psychological barriers by offering a treatment option that does not require physical attendance at a clinic and provides privacy and anonymity (Khadjesari et al., 2015). These conditions could make e-alcohol therapy particularly beneficial for individuals with concurrent problematic alcohol use and symptoms of anxiety or/and depression.

In a randomized controlled trial, we compared a proactive e-alcohol therapy intervention with face-to-face alcohol therapy (standard care), targeting individuals with problematic alcohol use who were not engaging in alcohol treatment. The therapy was proactive as the therapist initiated the first therapy session. The trial found that proactive e-alcohol therapy, compared to standard care, was more effective in increasing treatment initiation and compliance, while being equally effective in reducing alcohol intake (Egan et al., 2024). Besides that study, the literature on e-alcohol therapy is scarce. A study by de Beurs et al. (2023) investigated alcohol treatment via video conference during COVID-19 social distancing and found it non-inferior to in-person treatment in clinical effectiveness. Furthermore, a feasibility study on group sessions conducted via video conference — with the therapist at a remote site — identified high levels of client satisfaction, good session attendance, and low attrition (Frueh et al., 2005).

Based on post hoc analyses of data from the randomized controlled trial of proactive e-alcohol therapy, the aim of the present investigation was twofold: First, to explore whether anxiety or/and depressive symptoms modify the effect of proactive e-alcohol therapy on treatment initiation, compliance, and alcohol intake. Second, to examine the impact of proactive e-alcohol therapy on anxiety or/and depressive symptoms compared to standard care.

## 2 Methods

### 2.1 Study design

The present study is based on post hoc analyses from a randomized controlled trial, which tested whether proactive e-alcohol therapy improved treatment initiation, treatment compliance, and alcohol intake in comparison to standard care. Participants were individually assigned in equal ratio to receive either standard care or proactive e-alcohol therapy and were followed up at 3- and 12-months post randomization. Data were collected at the National Institute of Public Health in Denmark and pre-registered with the Danish Data Protection Agency (J.nr. 2015–57-0008). The trial was reviewed by the Capital Region’s Committee on Health Research Ethics in Denmark (Committee C, J.nr. H-16035716) but did not require formal approval under Danish law since it did not involve invasive procedures, medical drugs, or equipment. Also, the trial was prospectively registered with ClinicalTrials.gov (NCT03116282). A more detailed description of the trial is reported elsewhere (Egan et al., 2024).

### 2.2 Participants

A total of 356 individuals with problematic alcohol use were enrolled in the trial, recruited through a project website from January 2018 to June 2020. The website referred to the treatment as counseling and emphasized the benefits of making changes in drinking habits. It also highlighted that the treatment was free and could be accessed anonymously. The trial design and its implications were described, and visitors to the website had the option to complete the Alcohol Use Disorders Identification Test (AUDIT) and receive standardized written feedback on the benefits of changing drinking habits. Information on age and sex was provided with this anonymous test. The site was promoted on the internet using ads on Google and Facebook, as well as on relevant alcohol-related websites.

The study included individuals with an AUDIT score of 8 or higher, aged 18 years or older, who had access to a personal computer, smartphone, or tablet with a functional camera, audio equipment, and an internet connection. Exclusion criteria were refusal to provide information about municipality of residence, phone number, and email address. Those excluded were informed of exclusion reasons and provided with details on where to get help.

### 2.3 Randomization and blinding

Upon registration, participants received an email with links to detailed participant information and the baseline questionnaire in which informed consent was obtained. Randomization took place upon completion of the baseline questionnaire (allocation ratio 1:1) (Egan et al., 2024). Participants were not blinded, and data analyses in this study were conducted without blinding.

### 2.4 Procedures

If allocated to proactive e-alcohol therapy, an alcohol therapist contacted the participant within three to five weekdays to schedule the first session. The standard procedure involved online therapy through video conferencing using Skype for Business, but a pragmatic approach allowed for sessions over the phone or, in rare instances, in person. The therapeutic content of the sessions was based on motivational interviewing and cognitive behavioral therapy. The individual participant's needs determined the number, frequency, and duration of sessions. Any need for pharmacological treatment was handled by the participant's general practitioner. Therapists were from Novaví, Denmark's largest provider of substance abuse treatment.

Participants assigned to standard care were provided with contact details for their local alcohol treatment clinic and were prompted to initiate contact. Standard treatment consisted of face-to-face alcohol therapy and, when appropriate, pharmacological treatment. Sessions were delivered by an alcohol therapist at the individual's local municipal outpatient clinic, with the number, frequency, and duration of sessions tailored to each participant. In Denmark, alcohol treatment is, by law, free of charge and must be made available to all citizens within 14 days of inquiry, regardless of problem severity.

### 2.5 Data collection

Data for this study were self-reported by participants and collected through online questionnaires sent to their emails using the web-based software SurveyXact by Ramboll, Denmark. At baseline, participants provided information on alcohol use, previous alcohol treatment, motivation for change, anxiety and depressive symptoms, quality of life, self-rated health, social and demographic characteristics, and other health behaviours. At follow-up, participants primarily gave information on their alcohol use, their use of and experience with therapy. Information on anxiety and depressive symptoms was provided only after three months. Email reminders were dispatched after 48 hours and one week. For the follow-up questionnaires, three additional attempts were made to reach non-responders by telephone. At the 3- and 12-month follow-up, 87 and 80 participants, respectively, were reached by telephone and had the questionnaire resent via email. In total, three follow-up questionnaires were completed over the phone.

### 2.6 Modifiers

Three modifiers were used in this study: anxiety symptoms, depressive symptoms, and combined anxiety *and* depressive symptoms. Anxiety symptoms were measured by the General Anxiety Disorder-2 Scale (GAD-2) and depressive symptoms by the Patient Health Questionnaire-2 (PHQ-2), which were combined into one questionnaire battery. Participants were asked how often, over the last 2 weeks, they experienced the following: 1. Feeling nervous, anxious or on edge; 2. Not being able to stop or control worrying (GAD-2); 3. Little interest or pleasure in doing things, 4. Feeling down, depressed, or hopeless (PHQ-2). The screening tools GAD-2 and PHQ-2 both range from a score of 0 to 6, with a score of ≥ 3 indicating moderate or severe symptoms. Anxiety *and* depressive symptoms were measured by combining GAD-2 and PHQ-2, with a total score of 12 and a score of ≥ 6 used as an indicator of moderate or severe symptoms. When used in combination, these screening tools are referred to as the Patient Health Questionnaire-4 (PHQ-4) (Löwe et al., 2010). The Danish versions of the scales were used.

### 2.7 Outcomes

The following primary outcomes were assessed 3 and 12 months after randomization: Initiation of treatment (defined as completion of one therapy session); treatment compliance (defined as completion of at least three therapy sessions); and total weekly alcohol intake in standard drinks, measured by Alcohol Timeline Followback (TLFB). Participants reported their daily consumption of standard drinks for the past week, starting with ‘yesterday’ and proceeding one day at a time. They specified their intake of beer, wine, and spirits. A standard drink was defined as 12 g of pure alcohol, equivalent to a 33 cl bottle of beer (4–5 % alcohol) or a 12 cl glass of wine (12 % alcohol). This definition and additional examples were provided with the TLFB in the questionnaire. Treatment initiation was derived from the question: ‘Have you currently had one or more sessions about your alcohol habits with an alcohol therapist?’. Treatment compliance was derived from questions on the number of completed sessions via video conference, telephone, and face-to-face. Anxiety symptoms, depressive symptoms, and anxiety *and* depressive symptoms were measured by GAD-2, PHQ-2, and PHQ-4, respectively, at 3 months post-randomization.

### 2.8 Statistical analysis

Analyses of dichotomous outcomes were conducted using logistic regression, while continuous outcomes were analyzed with negative binomial regression, adjusting for baseline values, such as alcohol intake in standard drinks per week. Interaction analyses were performed to assess whether moderate-severe anxiety or/and depressive symptoms modified the differences between the intervention and control groups. Additional analyses of the modifying impact of anxiety or/and depressive symptoms as continuous scores were performed to ensure that the binary categorization of symptoms did not lose information. All analyses were carried out in Stata version 18. Results were computed for available cases.

Sensitivity analyses employing the intention-to-treat principle were conducted for primary outcomes to verify result robustness. Following this principle, missing values were accounted for using multiple imputations by chained equations (m = 40 imputations) (Spratt et al., 2010). Data were analyzed using the mi estimate command in Stata, which performs the estimation for each imputed dataset individually and then combines the results according to Rubin’s rules. Imputation was done separately for each arm. The imputation procedure included variables pre-hypothesized to potentially predict missing information (age, sex, education, baseline information: AUDIT score, alcohol intake, depression and anxiety, readiness to change, cohabitation status).

## 3 Results

**Fig. 1:**
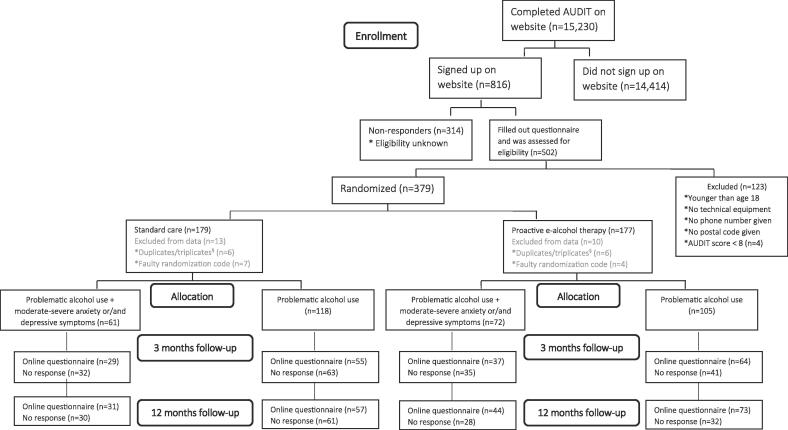
Participant flow in the trial. ^§^ Participants enrolled in the trial more than once.

Among the 502 individuals who filled out the baseline questionnaire and were assessed for eligibility, 379 (75 %) were randomly assigned to either proactive e-alcohol therapy (n = 187) or standard care (n = 192) between Jan 22, 2018, and Jun 29, 2020 (Fig. 1). However, the final sample for analysis included a total of 356 participants: 179 in the proactive e-alcohol therapy group and 177 in the standard care group. During data cleaning, 23 participants were removed from the dataset because of duplicates/triplicates and a faulty randomization code, which included participants who did not meet the technical equipment criteria. Duplicates/triplicates refer to participants enrolled in the trial more than once, which occurred due to open enrollment and were filtered out based on email address. A more thorough description of the participant flow is reported elsewhere (Egan et al., 2024).

Demographic and behavioural characteristics were balanced in the two groups at baseline and is shown in more detail elsewhere (Egan et al., 2024). Of all participants, 170 (48 %) were female. The median age was 46 (interquartile range 36, 56). 260 (73 %) had ≥ 13 years of education and a corresponding proportion were employed. Among participants, the median weekly alcohol intake was 28 drinks, the median AUDIT score was 20, and 54 % had an AUDIT score of 20 or more which indicates a high risk of alcohol dependence. In total, 26 % had previous experience with municipal alcohol treatment or treatment provided by a general practitioner. Participants were highly motivated to change as reflected by a high readiness to change score. Table 1 shows the distribution of anxiety or/and depressive symptoms among participants in both groups at baseline. On the PHQ-4 scale, 37 % showed moderate-severe symptoms. In the proactive e-alcohol therapy group, the proportion with no symptoms of anxiety and depression and moderate-severe symptoms was slightly higher than in the standard care group (Table 1). This was also the case for the GAD-2 scale measuring symptoms of anxiety and the PHQ-2 scale measuring symptoms of depression.

**Table 1:** Anxiety and depressive symptoms among participants at baseline. Values are number (%) for categorical variables and median (interquartile range 25, 75) for continuous variables.

	Standard care (n = 179)	E-alcohol therapy (n = 177)	All (n = 356)
**Anxiety** (GAD-2 score)			
No (0)	31 (17)	35 (20)	66 (19)
Mild (1–2)	78 (44)	68 (38)	146 (41)
Moderate or severe (3–6)	70 (39)	74 (42)	144 (40)
*Median*	2 (1, 4)	2 (1, 4)	2 (1, 4)
**Depression** (PHQ-2 score)			
No (0)	28 (16)	35 (20)	63 (18)
Mild (1–2)	89 (50)	70 (40)	159 (45)
Moderate or severe (3–6)	62 (34)	72 (41)	134 (38)
*Median*	2 (1, 3)	2 (1, 4)	2 (1, 4)
**Anxiety and depression** (PHQ-4 score)			
No (0)	17 (9)	25 (14)	42 (12)
Very mild (1–2)	33 (18)	21 (12)	54 (15)
Mild (3–5)	68 (38)	59 (33)	127 (36)
Moderate or severe (6–12)	61 (34)	72 (41)	133 (37)
*Median*	4 (2, 7)	4 (2, 7)	4 (2, 7)

Table 2 shows the modifying impact of moderate-severe anxiety or/and depressive symptoms on the effect of proactive e-alcohol therapy vs. standard care on treatment initiation and treatment compliance at 3- and 12-month follow-up. Initiation of treatment at 3-month follow-up was higher in the proactive e-alcohol therapy group compared to standard care, both among participants with moderate-severe symptoms of anxiety and depression and those with no moderate-severe symptoms, with no significant interaction between intervention group and moderate-severe anxiety and depressive symptoms (p = 0.64). Also, for treatment compliance at 3-month follow-up, participants in the proactive e-alcohol therapy group were more likely to have completed at least three therapy sessions compared to participants in the standard care group, both among participants with moderate-severe symptoms of anxiety and depression and those with no moderate-severe symptoms, with no significant interaction between intervention group and moderate-severe anxiety and depressive symptoms (p = 0.40) (Table 2). Results at 12-month follow-up were comparable. Similar results were observed in the intention to treat analyses, details are provided in Sup. Table 1, Table 2. Also, results were robust to the modifying impact of continuous symptom scores (Sup. Table S3).

**Table 2:** Modifying impact of moderate-severe anxiety or/and depressive symptoms on the effect of proactive e-alcohol therapy vs. standard care on treatment initiation^a^ and treatment compliance^b^ at 3- and 12-month follow-up.

		Standard care	E-alcohol therapy		
		% (N/total N)	% (N/total N)	Odds ratio (95 % CI)	Interaction p value^c^
3 months follow-up					
**Treatment initiation**					
Anxiety symptoms (GAD-2 score)					0.55
	No (0–2)	46 (23/50)	87 (55/63)	8.1 (3.2 to 20.4)	
	Yes (3–6)	71 (24/34)	92 (35/38)	4.9 (1.2 to 19.5)	
Depressive symptoms (PHQ-2 score)					0.97
	No (0–2)	55 (29/53)	89 (55/62)	6.5 (2.5 to 16.9)	
	Yes (3–6)	58 (18/31)	90 (35/39)	6.3 (1.8 to 22.2)	
Anxiety and depressive symptoms (PHQ-4 score)					0.64
	No (0–5)	51 (28/55)	86 (55/64)	5.9 (2.4 to 14.2)	
	Yes (6–12)	66 (19/29)	95 (35/37)	9.2 (1.8 to 46.4)	
**Treatment compliance**					
Anxiety symptoms (GAD-2 score)					0.88
	No (0–2)	40 (20/50)	73 (45/62)	4.0 (1.8 to 8.8)	
	Yes (3–6)	42 (14/33)	76 (29/38)	4.4 (1.6 to 12.1)	
Depressive symptoms (PHQ-2 score)					0.21
	No (0–2)	44 (23/52)	71 (43/61)	3.0 (1.4 to 6.5)	
	Yes (3–6)	35 (11/31)	79 (31/39)	7.0 (2.4 to 20.5)	
Anxiety and depressive symptoms (PHQ-4 score)					0.40
	No (0–5)	43 (23/54)	71 (45/63)	3.4 (1.6 to 7.3)	
	Yes (6–12)	38 (11/29)	78 (29/37)	5.9 (2.0 to 17.5)	
12 months follow-up					
**Treatment initiation**					
Anxiety symptoms (GAD-2 score)					0.45
	No (0–2)	58 (30/52)	85 (61/72)	4.1 (1.7 to 9.5)	
	Yes (3–6)	78 (28/36)	89 (40/45)	2.3 (0.7 to 7.7)	
Depressive symptoms (PHQ-2 score)					0.55
	No (0–2)	63 (37/59)	86 (63/73)	3.7 (1.6 to 8.8)	
	Yes (3–6)	72 (21/29)	86 (38/44)	2.4 (0.7 to 7.9)	
Anxiety and depressive symptoms (PHQ-4 score)					0.97
	No (0–5)	63 (36/57)	85 (62/73)	3.3 (1.4 to 7.6)	
	Yes (6–12)	71 (22/31)	89 (39/44)	3.2 (0.9 to 10.7)	
**Treatment compliance**					
Anxiety symptoms (GAD-2 score)					0.83
	No (0–2)	49 (25/51)	77 (51/66)	3.5 (1.6 to 7.8)	
	Yes (3–6)	59 (19/32)	86 (36/42)	4.1 (1.3 to 12.5)	
Depressive symptoms (PHQ-2 score)					1.0
	No (0–2)	51 (28/55)	79 (53/67)	3.7 (1.7 to 8.1)	
	Yes (3–6)	57 (16/28)	83 (34/41)	3.6 (1.2 to 11.0)	
Anxiety and depressive symptoms (PHQ-4 score)					0.58
	No (0–5)	52 (28/54)	78 (52/67)	3.2 (1.5 to 7.1)	
	Yes (6–12)	55 (16/29)	85 (35/41)	4.7 (1.5 to 14.7)	

a Completion of one therapy session.

b Completion of at least three therapy sessions.

c Interaction between intervention group and symptoms.

Table 3 shows the modifying impact of moderate-severe symptoms of anxiety or/and depressive symptoms on the effect of proactive e-alcohol therapy vs. standard care on alcohol intake (standard drinks/week) at 3- and 12-month follow-up. At 3-month follow-up, participants in the proactive e-alcohol therapy group had a lower weekly alcohol intake compared to those in standard care both among those with and without moderate-severe symptoms of anxiety or/and depression at baseline. There were no significant interactions between intervention group and moderate-severe anxiety or/and depressive symptoms. For example, at 3-month follow-up, participants with no moderate-severe anxiety and depressive symptoms in the proactive e-alcohol therapy group drank on average 15.6 drinks a week and those in standard care drank 20.9 drinks a week. Among those with moderate-severe anxiety and depressive symptoms, participants in the proactive e-alcohol therapy group drank on average 12.7 drinks a week, and those in the standard care group, 18.5 drinks a week. The p value for interaction was 0.86. At 12-month follow-up, there was no significant difference in alcohol intake among participants in the proactive e-alcohol therapy group and those in standard care, neither among participants with moderate-severe symptoms of anxiety or/and depression, nor among those without. Similar results for alcohol intake were observed in the intention to treat analyses, details are provided in Sup. Table 2. Also, results were robust to the modifying impact of continuous symptom scores (Supplementary Figures S1-S3).

**Table 3:** Modifying impact of moderate-severe anxiety or/and depressive symptoms on the effect of proactive e-alcohol therapy vs. standard care on alcohol intake (standard drinks/week) at 3- and 12-month follow-up.

		Standard care	E-alcohol therapy		
		Mean	Mean	Difference in means (95 % CI)^a^	Interaction p value^b^
3 months follow-up					
Anxiety symptoms (GAD-2 score)					0.95
	No (0–2)	20.8	15.1	−5.7 (−13.4 to 2.0)	
	Yes (3–6)	17.9	13.2	−4.7 (−15.0 to 5.6)	
Depressive symptoms (PHQ-2 score)					0.48
	No (0–2)	19.1	15.5	−3.6 (−10.4 to 3.1)	
	Yes (3–6)	20.8	11.7	−9.2 (−21.3 to 2.9)	
Anxiety and depressive symptoms (PHQ-4 score)					0.86
	No (0–5)	20.9	15.6	−5.3 (−12.9 to 2.3)	
	Yes (6–12)	18.5	12.7	−5.8 (−16.5 to 4.8)	
12 months follow-up					
Anxiety symptoms (GAD-2 score)					0.85
	No (0–2)	15.2	15.7	0.5 (−8.0 to 9.0)	
	Yes (3–6)	13.8	14.1	0.3 (−8.8 to 9.5)	
Depressive symptoms (PHQ-2 score)					1.0
	No (0–2)	13.6	14.6	1.1 (−6.5 to 8.7)	
	Yes (3–6)	15.4	14.1	−1.3 (−11.1 to 8.5)	
Anxiety and depressive symptoms (PHQ-4 score)					0.90
	No (0–5)	14.5	15.2	0.7 (−7.5 to 8.9)	
	Yes (6–12)	14.7	14.6	−0.1 (−9.5 to 9.4)	

a Adjusted by weekly alcohol intake at baseline.

b Interaction between intervention group and symptoms.

At 3-month follow-up, no difference was observed between the proportion of participants with moderate-severe anxiety or/and depressive symptoms in the two intervention groups: the proportion of participants with moderate-severe symptoms was approximately halved (Table 4). Repeating the analyses with anxiety or/and depressive symptoms modelled on a continuous scale did not change the findings (Sup. Table S4).

**Table 4:** Impact of proactive e-alcohol therapy vs. standard care on moderate-severe anxiety or/and depressive symptoms at 3-month follow-up.

	Standard care	E-alcohol therapy				
	% (N/total N)	% (N/total N)	Difference in % (95 % CI)^a^	P value	Odds ratio (95 % CI)^a^	P value
**Anxiety symptoms** (GAD-2 score ≥ 3)						
Available cases (AC)	19 (16/84)	19 (19/100)	0 (−11 to 11)	0.97	1.0 (0.5 to 2.1)	0.97
Intention to treat (ITT)	23 (41/179)	20 (35/177)	−3 (−15 to 8)	0.59	0.8 (0.4 to 1.7)	0.64

**Depressive symptoms** (PHQ-2 score ≥ 3)						
AC	19 (16/84)	16 (16/100)	−3 (−14 to 7)	0.52	0.8 (0.3 to 1.7)	0.59
ITT	21 (38/179)	17 (30/177)	−6 (−16 to 5)	0.30	0.7 (0.3 to 1.4)	0.29

**Anxiety and depressive symptoms** (PHQ-4 score ≥ 6)						
AC	18 (15/84)	12 (12/100)	−10 (–22 to 2)	0.11	0.5 (0.2 to 1.2)	0.11
ITT	21 (38/179)	13 (23/177)	−11 (–23 to 2)	0.09	0.5 (0.2 to 1.1)	0.09


a Adjusted by symptoms at baseline.

In Table 5 it appears that the mean number of video and telephone sessions in the proactive e-alcohol therapy group were similar among participants with and without moderate-severe symptoms of anxiety and depression at both 3- and 12-month follow-up.

**Table 5:** Number of therapy sessions completed by participants with and without moderate-severe anxiety and depressive symptoms (PHQ-4 score ≥ 6) in the proactive e-alcohol therapy group, split by type of communication channel.

	E-alcohol therapy						
3 months follow-up							
	Problematic alcohol use + anxiety and depressive symptoms				Problematic alcohol use		
	Sessions (N)	Participants (N)	Mean		Sessions (N)	Participants (N)	Mean
Video sessions	84	28	3.0		137	35	3.9
Telephone sessions	54	21	2.6		75	29	2.6
Face-to-face sessions	10	4	2.5		20	4	5
Therapy sessions, total	148				232		

**12 months follow-up**							

	**Problematic alcohol use + anxiety and depressive symptoms**				**Problematic alcohol use**		

**Sessions (N)**	**Participants (N)**	**Mean**		**Sessions (N)**	**Participants (N)**	**Mean**

Video sessions	175	16	10.9		286	30	9.5
Telephone sessions	120	15	8		156	20	7.8
Face-to-face sessions	45	3	15		39	5	7.8
Therapy sessions, total	340				481		

## 4 Discussion

The aim of this study was twofold. Firstly, we explored whether anxiety or/and depressive symptoms modified the effect of proactive e-alcohol therapy on treatment initiation, compliance, and alcohol intake. No significant interaction was found between moderate-severe anxiety or/and depressive symptoms and therapy group regarding initiation of treatment, compliance to treatment, or alcohol intake at 3- and 12-month follow-up. These results suggest that proactive e-alcohol therapy is similarly effective for individuals with problematic alcohol use, regardless of co-occurring anxiety or/and depressive symptoms. Previous studies on traditional face-to-face addiction treatment have found anxiety and depressive symptoms to pose a barrier to successful treatment (Buitelaar et al., 2015, Farris et al., 2012, van Hagen et al., 2019). Consequently, proactive e-alcohol therapy might be a suitable treatment alternative for this subgroup of individuals with problematic alcohol use and co-occurring symptoms of anxiety or/and depression.

Secondly, we examined the impact of proactive e-alcohol therapy on anxiety or/and depressive symptoms compared to standard care at 3-month follow-up. No significant difference in the proportion of participants with moderate-severe anxiety or/and depressive symptoms was found between the two intervention groups. As the number of participants experiencing moderate-severe symptoms was halved, this finding indicates that psychosocial alcohol therapy itself might have a beneficial impact on anxiety and depressive symptoms, independent of the therapy’s communication channel. This adds to the existing literature highlighting the effectiveness of psychosocial therapy for anxiety and depression (WHO, 2023) and suggests potential benefits of interventions that address overlapping symptoms of multiple disorders. Current recommendations advocate for specific treatment of co-occurring mental disorders alongside alcohol treatment, as this approach improves prognosis (Baker et al., 2012; Hobbs et al., 2011).

At baseline, there was a slightly higher number of participants with moderate-severe symptoms in the proactive e-alcohol therapy group compared to standard care. This imbalance could introduce confounding, potentially influencing our findings. Also, it is important in the interpretation of our findings to note, that only a few studies have been conducted on which screening instruments are applicable for identifying anxiety and depression among people with problematic alcohol use (Andersen et al., 2023), and the GAD-2, PHQ-2, and PHQ-4 scales are indicative of symptoms only and not diagnostic tools. For some individuals entering alcohol treatment while experiencing symptoms of anxiety or/and depression, these symptoms may resolve during treatment, while they may persist or worsen in others, which can increase the risk of negative outcomes. Rabinowitz et al. (2023) found that symptom trajectories of anxiety and depression were linked to treatment attrition among individuals in alcohol treatment, and these trajectories were associated with patient demographics and substance use. For example, they found that women were more prone to experience persistent anxiety and depressive symptoms. The group with concurrent problematic alcohol use and anxiety or/and depressive symptoms in this study is likely heterogeneous in terms of symptom trajectories, which is not captured by baseline measurements with GAD-2, PHQ-2, and PHQ-4, potentially masking subgroups that exhibit varying degrees of response to the intervention. The use of the screening tools may further be limiting, as the tools measure only a few core anxiety and depressive symptoms. Participants with other symptoms may not have been detected. However, GAD-2, PHQ-2, and PHQ-4 have been validated in several studies, all of which show they have good sensitivity and specificity for detecting anxiety and depression (Kroenke et al., 2010, Löwe et al., 2010).

Participants in this trial were highly motivated to change, as indicated by a high readiness to change score at baseline (Egan et al., 2024). The proactive phone call made by the therapist to arrange the first e-alcohol therapy session may have tapped into this motivation among participants in the intervention group. Additionally, this proactive component could have been particularly valuable for participants with co-occurring anxiety or/and depressive symptoms, who could have significantly benefited from having the therapist take the lead in initiating the first session. If this were the case, it would likely have been reflected in the initiation outcome. Similarly, the proactive email containing contact information for a local clinic in the standard care group might have also benefitted the participants with co-occurring anxiety or/and depressive symptoms, potentially impacting the initiation outcome. Typically, people would need to locate this information on their own.

This study makes an important contribution to the existing literature by exploring the relationship between proactive e-alcohol therapy and symptoms of anxiety or/and depression among individuals with problematic alcohol use. It builds on a large trial with a 12-month long-term follow-up, effectively engaging a population distinct from those typically seen in alcohol treatment (for example, participants were more frequently female, employed, and had lower weekly alcohol consumption) (Egan et al., 2024). These characteristics are important for assessing the generalizability of the study's results. It is also important to note that this study population was self-motivated to seek treatment and highly motivated to change.

The study has several limitations: Firstly, it was not adequately powered to detect interactions between the intervention group and anxiety/depressive symptoms. Building on this, the impact of proactive e-alcohol therapy on anxiety or/and depressive symptoms was also not further explored in subgroups defined by baseline symptoms. Secondly, the findings rely on self-reports. Thirdly, there was a substantial number of participants lost to follow-up in the trial, with varying rates between the two intervention groups. Result robustness was verified by supplementary analyses employing the intention-to-treat principle.

## 5 Conclusions

In conclusion, this study suggests that proactive alcohol therapy is effective for individuals with problematic alcohol use, regardless of co-occurring anxiety or/and depressive symptoms. Moreover, it finds that proactive e-alcohol therapy and standard care have a similar impact on reducing symptoms of anxiety and depression. It is crucial to gain a better understanding of the severity of these concurrent problems and how severity may impact the effectiveness of proactive e-alcohol therapy.

## Role of funding source

The study was funded by TrygFonden. The funders of the study had no role in study design, data collection, data analysis, data interpretation, or writing of this paper.

## Data sharing

Due to data privacy regulations, data generated during this study are not publicly accessible. Access to anonymized data may be granted upon evaluation by the principal investigator and the trial management group. Additionally, project-related documents will be available upon request. All inquiries should be directed to the corresponding author.

## Credit authorship contribution statement

**Kia Kejlskov Egan:** Writing – original draft, Project administration, Investigation, Formal analysis, Data curation, Conceptualization. **Veronica Pisinger:** Writing – review & editing, Writing – original draft, Validation, Formal analysis, Conceptualization. **Ulrik Becker:** Writing – review & editing, Supervision, Funding acquisition. **Janne Schurmann Tolstrup:** Conceptualization, Formal analysis, writing - review and editing, Supervision, Funding Acquisition.

## Declaration of competing interest

During the study period, the National Institute of Public Health, University of Southern Denmark, received a grant from Novaví to evaluate their other treatment offers. This evaluation was conducted by KKE and UB. UB had travel expenses covered by Novaví for his participation in a meeting on the Faroe Islands about local alcohol treatment opportunities. UB also received an honorarium for giving a lecture on evidence-based alcohol treatment for Novaví. All authors declare no competing interests.

## Data Availability

Data can be made available on request.
