# Supplementary material for: Exploring the relationship between proactive e-alcohol therapy and symptoms of anxiety or/and depression: Post-hoc analyses from a randomized controlled trial
Source: Addict Behav Rep. 2024 Dec 5;21:100576. doi: 10.1016/j.abrep.2024.100576 (PMC11696633; doi:10.1016/j.abrep.2024.100576)
Supplement: Supplementary Data 1 [file mmc1.docx]

**Supplementary tables**

**Table S1** Modifying impact of moderate-severe anxiety or/and depressive symptoms on the effect of proactive e-alcohol therapy vs. standard care on treatment initiation^a^ and treatment compliance^b^ at 3- and 12-month follow-up. Intention to treat analyses.

|  | | | Standard care   (n=179) | E-alcohol therapy  (n=177) |  |  | |
| --- | --- | --- | --- | --- | --- | --- | --- |
|  |  |  | % (N/total N) | % (N/total N) | Odds ratio (95 % CI) | Interaction p value^c^ | |
| 3 months follow-up | | |  |  |  | |  |
| **Treatment initiation** | | |  |  |  | |  |
| Anxiety symptoms (GAD-2 score) | | |  |  |  | | 0.67 |
|  | No (0-2) | | 46 (50/109) | 85 (88/103) | 7.4 (2.7 to 19.6) | |  |
|  | Yes (3-6) | | 66 (46/70) | 91 (67/74) | 5.0 (1.2 to 21.4) | |  |
| Depressive symptoms (PHQ-2 score) | | |  |  |  | | 0.76 |
|  | No (0-2) | | 51 (60/117) | 88 (92/105) | 6.9 (2.6 to 18.9) | |  |
|  | Yes (3-6) | | 58 (36/62) | 88 (63/72) | 5.4 (1.4 to 20.5) | |  |
| Anxiety and depressive symptoms (PHQ-4 score) | | |  |  |  | | 0.97 |
|  | No (0-5) | | 49 (58/118) | 86 (90/105) | 6.2 (2.5 to 15.8) | |  |
|  | Yes (6-12) | | 62 (38/61) | 90 (65/72) | 6.5 (1.3 to 31.6) | |  |
| **Treatment compliance** | | |  |  |  | |  |
| Anxiety symptoms (GAD-2 score) | | |  |  |  | | 0.60 |
|  | No (0-2) | | 42 (46/109) | 71 (73/103) | 3.5 (1.6 to 7.3) | |  |
|  | Yes (3-6) | | 40 (28/70) | 77 (57/74) | 4.9 (1.7 to 14.2) | |  |
| Depressive symptoms (PHQ-2 score) | | |  |  |  | | 0.25 |
|  | No (0-2) | | 44 (52/117) | 70 (74/105) | 3.0 (1.4 to 6.3) | |  |
|  | Yes (3-6) | | 35 (22/62) | 78 (56/72) | 6.8 (2.2 to 20.8) | |  |
| Anxiety and depressive symptoms (PHQ-4 score) | | |  |  |  | | 0.33 |
|  | No (0-5) | | 44 (52/118) | 70 (74/105) | 3.1 (1.5 to 6.6) | |  |
|  | Yes (6-12) | | 38 (23/61) | 78 (56/72) | 6.0 (2.1 to 17.6) | |  |
| 12 months follow-up | | |  |  |  | |  |
| **Treatment initiation** | | |  |  |  | |  |
| Anxiety symptoms (GAD-2 score) | | |  |  |  | | 0.42 |
|  | No (0-2) | | 57 (62/109) | 83 (86/103) | 3.9 (1.6 to 9.2) | |  |
|  | Yes (3-6) | | 76 (53/70) | 88 (65/74) | 2.2 (0.8 to 6.3) | |  |
| Depressive symptoms (PHQ-2 score) | | |  |  |  | | 0.37 |
|  | | No (0-2) | 62 (72/117) | 87 (91/105) | 4.0 (1.7 to 9.5) | |  |
|  | | Yes (3-6) | 71 (44/62) | 83 (60/72) | 2.1 (0.7 to 6.4) | |  |
| Anxiety and depressive symptoms (PHQ-4 score) | | |  |  |  | | 0.57 |
|  | | No (0-5) | 61 (72/118) | 85 (89/105) | 3.6 (1.6 to 8.2) | |  |
|  | | Yes (6-12) | 72 (44/61) | 86 (62/72) | 2.5 (0.9 to 7.0) | |  |
| **Treatment compliance** | | |  |  |  | |  |
| Anxiety symptoms (GAD-2 score) | | |  |  |  | | 0.71 |
|  | No (0-2) | | 50 (54/109) | 76 (78/103) | 3.1 (1.4 to 6.8) | |  |
|  | Yes (3-6) | | 59 (41/70) | 84 (62/74) | 4.0 (1.3 to 12.2) | |  |
| Depressive symptoms (PHQ-2 score) | | |  |  |  | | 0.91 |
|  | | No (0-2) | 50 (59/117) | 78 (82/105) | 3.5 (1.5 to 8.0) | |  |
|  | | Yes (3-6) | 58 (36/62) | 81 (58/72) | 3.2 (1.1 to 9.4) | |  |
| Anxiety and depressive symptoms (PHQ-4 score) | | |  |  |  | | 0.81 |
|  | | No (0-5) | 51 (60/118) | 76 (80/105) | 3.2 (1.4 to 7.0) | |  |
|  | | Yes (6-12) | 57 (35/61) | 83 (60/72) | 3.7 (1.3 to 11.0) | |  |

^a^ Completion of one therapy session.

^b^ Completion of at least three therapy sessions.

^c^ Interaction between intervention and symptoms.

**Table S2** Modifying impact of moderate-severe anxiety or/and depressive symptoms on the effect of proactive e-alcohol therapy vs. standard care on weekly alcohol intake (standard drinks/week) at 3- and 12-month follow-up. Intention to treat analyses.

|  | | | Standard care (n=179) | E-alcohol therapy (n=177) |  |  |
| --- | --- | --- | --- | --- | --- | --- |
|  |  |  | Mean | Mean | Difference in means (95% CI)^a^ | Interaction p value ^b^ |
| 3 months follow-up | | |  |  |  |  |
| Anxiety symptoms (GAD-2 score) | | |  |  |  | 0.93 |
|  | | No (0-2) | 21.0 | 14.1 | -7.0 (-13.7 to -0.2) |  |
|  | | Yes (3-6) | 20.8 | 14.4 | -6.4 (-16.2 to 3.4) |  |
| Depressive symptoms (PHQ-2 score) | | |  |  |  | 0.75 |
|  | | No (0-2) | 19.1 | 14.6 | -4.5 (-11.0 to 2.0) |  |
|  | | Yes (3-6) | 23.1 | 12.9 | -10.2 (-20.4 to 0.0) |  |
| Anxiety and depressive symptoms (PHQ-4 score) | | |  |  |  | 0.99 |
|  | | No (0-5) | 20.8 | 14.7 | -6.1 (-12.9 to 0.75) |  |
|  | | Yes (6-12) | 21.7 | 13.6 | -8.1 (-18.1 to 2.0) |  |
| 12 months follow-up | | |  |  |  |  |
| Anxiety symptoms (GAD-2 score) | | |  |  |  | 0.91 |
|  | No (0-2) | | 15.5 | 14.3 | -1.2 (-8.7 to 6.3) |  |
|  | Yes (3-6) | | 14.2 | 13.9 | -0.3 (-8.1 to 7.4) |  |
| Depressive symptoms (PHQ-2 score) | | |  |  |  | 0.71 |
|  | No (0-2) | | 14.1 | 13.9 | -0.2 (-7.2 to 6.9) |  |
|  | Yes (3-6) | | 16.0 | 14.0 | -2.1 (-11.4 to 7.3) |  |
| Anxiety and depressive symptoms (PHQ-4 score) | | |  |  |  | 0.79 |
|  | No (0-5) | | 14.8 | 14.1 | -0.7 (-7.9 to 6.5) |  |
|  | Yes (6-12) | | 15.3 | 14.2 | -1.0 (-9.7 to 7.6) |  |

^a^ Adjusted by weekly alcohol intake at baseline.
^b^ Interaction between intervention group and symptoms.

**Table S3** Modifying impact of anxiety or/and depressive symptoms (continuous scores) on the effect of proactive e-alcohol therapy on treatment initiation^a^ and treatment compliance^b^ at 3- and 12-month follow-up. Available case analyses.

|  | Proactive e-alcohol therapy odds ratio  (95 % CI) | Symptoms  odds ratio  (95 % CI) | Interaction odds ratio  (95 % CI) | Interaction p value^c^ |
| --- | --- | --- | --- | --- |
| 3 months follow-up |  |  |  |  |
| **Anxiety symptoms**  (GAD-2 score 0-6) |  |  |  |  |
| Treatment initiation | 5.9 (1.8 to 19.4) | 1.2 (1.0 to 1.6) | 1.1 (0.7 to 1.7) | 0.7 |
| Treatment compliance | 3.6 (1.3 to 10.1) | 1.0 (0.8 to 1.3) | 1.1 (0.7 to 1.5) | 0.7 |
|  |  |  |  |  |
| **Depressive symptoms**  (PHQ-2 score 0-6) |  |  |  |  |
| Treatment initiation | 6.3 (1.9 to 21.1) | 1.2 (0.9 to 1.5) | 1.0 (0.6 to 1.7) | 0.9 |
| Treatment compliance | 3.4 (1.2 to 9.8) | 1.1 (0.8 to 1.4) | 1.1 (0.8 to 1.6) | 0.6 |
|  |  |  |  |  |
| **Anxiety and depressive symptoms**  (PHQ-4 score 0-12) |  |  |  |  |
| Treatment initiation | 6.0 (1.7 to 21.4) | 1.1 (1.0 to 1.3) | 1.0 (0.8 to 1.4) | 0.8 |
| Treatment compliance | 3.3 (1.1 to 9.9) | 1.0 (0.9 to 1.1) | 1.1 (0.9 to 1.3) | 0.6 |
|  |  |  |  |  |
| 12 months follow-up |  |  |  |  |
| **Anxiety symptoms**  (GAD-2 score 0-6) |  |  |  |  |
| Treatment initiation | 3.5 (1.2 to 9.9) | 1.3 (1.0 to 1.6) | 1.0 (0.7 to 1.5) | 1.0 |
| Treatment compliance | 2.8 (1.0 to 7.3) | 1.1 (0.9 to 1.4) | 1.2 (0.8 to 1.7) | 0.4 |
|  |  |  |  |  |
| **Depressive symptoms**  (PHQ-2 score 0-6) |  |  |  |  |
| Treatment initiation | 3.6 (1.2 to 10.4) | 1.1 (0.9 to 1.5) | 1.0 (0.7 to 1.4) | 0.9 |
| Treatment compliance | 3.4 (1.3 to 9.2) | 1.1 (0.9 to 1.4) | 1.1 (0.7 to 1.5) | 0.8 |
|  |  |  |  |  |
| **Anxiety and depressive symptoms**  (PHQ-4 score 0-12) |  |  |  |  |
| Treatment initiation | 3.6 (1.2 to 10.9) | 1.1 (1.0 to 1.3) | 1.0 (0.8 to 1.2) | 0.9 |
| Treatment compliance | 2.9 (1.0 to 8.2) | 1.1 (0.9 to 1.2) | 1.1 (0.9 to 1.3) | 0.5 |

^a^ Completion of one therapy session.

^b^ Completion of at least three therapy sessions.

^c^ Interaction between intervention and symptoms.

**Table S4** Impact of proactive e-alcohol therapy (vs. standard care) on anxiety or/and depressive symptoms at 3-month follow-up.

|  | Standard care | E-alcohol therapy |  |  |
| --- | --- | --- | --- | --- |
|  | Mean | Mean | Difference in means  (95% CI)^a^ | P value |
| **Anxiety symptoms**  (GAD-2 score 0-6) |  |  |  |  |
| Available case (AC) | 1.5 | 1.3 | -0.2 (-0.6 to 0.2) | 0.4 |
| Intention to treat (ITT) | 1.6 | 1.4 | -0.3 (-0.7 to 0.2) | 0.3 |
|  | | | | |
| **Depressive symptoms**  (PHQ-2 score 0-6) |  |  |  |  |
| AC | 1.4 | 1.3 | -0.1 (-0.5 to 0.3) | 0.7 |
| ITT | 1.5 | 1.3 | -0.2 (-0.6 to 0.3) | 0.5 |
|  | | | | |
| **Anxiety and depressive symptoms**  (PHQ-4 score 0-12) |  |  |  |  |
| AC | 2.9 | 2.6 | -0.3 (-1.0 to 0.5) | 0.5 |
| ITT | 3.1 | 2.7 | -0.4 (-1.2 to 0.4) | 0.3 |
|  | | | | |

^a^ Adjusted by symptoms at baseline.

**Figure S1** Modifying impact of anxiety symptoms (GAD-2 score 0-6) on the effect of proactive e-alcohol therapy vs. standard care on weekly alcohol intake (standard drinks/week) at 3-month follow-up. Available case analyses.

**Figure S2** Modifying impact of depressive symptoms (PHQ-2 score 0-6) on the effect of proactive e-alcohol therapy vs. standard care on weekly alcohol intake (standard drinks/week) at 3-month follow-up. Available case analyses.

**Figure S3** Modifying impact of anxiety and depressive symptoms (PHQ-4 score 0-12) on the effect of proactive e-alcohol therapy vs. standard care on weekly alcohol intake (standard drinks/week) at 3-month follow-up. Available case analyses.
